# Cellular signal–responsive m^5^C editing by an inducible split CRISPR platform

**DOI:** 10.1261/rna.081049.126

**Published:** 2026-11

**Authors:** Chanjuan Dong, Ying Xu, Kellie Wu, Nathan Mu, Satendra Kumar, Fu-Sen Liang

**Affiliations:** 1 Department of Chemistry, Case Western Reserve University, Cleveland, Ohio 44106, USA; 2 Department of Psychology, Yale University, New Haven, Connecticut 06510, USA

**Keywords:** CRISPR, split dCas13b, m^5^C, ABA prodrug, CIP

## Abstract

RNA 5-methylcytidine (m^5^C) modification plays an essential role in regulating RNA metabolism and functions in cellular processes. Tools to achieve temporal and transcript-specific m^5^C editing for functional studies are still limited. Furthermore, methods enabling m^5^C editing triggered by specific cellular signals to study m^5^C functions are currently lacking. Here, we present a temporally and conditionally controlled m^5^C writing platform engineered through integrating abscisic acid (ABA)-mediated chemically induced proximity with split-dCas13b-NSUN2/NSUN6 technology. This system enables the writing of m^5^C by reconstituting the split dCas13b-based m^5^C editing complex at the guide RNA (gRNA)-targeted RNA transcript sites under the control of the inducer ABA. The deposition of m^5^C is inducible, reversible and selective. The deposited m^5^C is biologically active and influences the stability of endogenous mRNA transcripts. Moreover, by incorporating ABA prodrugs, the m^5^C writing can be triggered by signals associated with distinct physiological or disease conditions (e.g., tumor microenvironment and senescence). This conditional m^5^C editing strategy provides a new programmable tool for studying m^5^C biology in a context-dependent manner and expands the repertoire of RNA modification editing technologies.

## INTRODUCTION

RNA modifications have been demonstrated to mediate various cellular functions under physiological and pathological conditions (Nachtergaele and He 2017; Roundtree et al. 2017; Wilkinson et al. 2022). It is known that the roles of RNA modifications are context dependent, which can vary based on RNA transcripts and the modification sites (Shi et al. 2019). m^5^C, a prevalent chemical modification occupying ∼0.03%–0.1% of all cytosines, has been identified in ribosomal RNA (rRNA), transfer RNA (tRNA), and messenger RNA (mRNA) by transcriptome-wide RNA-seq studies (Edelheit et al. 2013; Wang et al. 2024). NOL/NOP2/NSUN family members as well as DNA methyltransferase, have been reported to catalyze the m^5^C deposition on mRNAs, while TET family proteins and ALKBH1 have been suggested to be responsible for m^5^C removal (Tan and Shi 2012; Chen et al. 2021; Guo et al. 2021; Jiang et al. 2025; Zhao et al. 2025). m^5^C has been shown to affect RNA biogenesis (Hussain et al. 2013; Cui et al. 2017; Liang et al. 2020), structure, stability (Gigova et al. 2014; Chen et al. 2019; Yang et al. 2019), and protein translation (Tuorto et al. 2012; Janin et al. 2019). Dysregulation of m^5^C has also been connected to several human diseases (Khosronezhad et al. 2015; Guo et al. 2020; Kong et al. 2020; Sun et al. 2020; Hu et al. 2021; Yang et al. 2023a,b).

Several clustered regularly interspaced short palindromic repeats (CRISPR)-based RNA modification editing tools have been reported to achieve transcript-specific editing, which mainly focused on m^6^ A editing (Liu et al. 2019; Li et al. 2020a; Wilson et al. 2020; Zhao et al. 2020; Xia et al. 2021). One m^5^C editing platform using dCasRx fused to the m^5^C writer and eraser has been reported to enable m^5^C editing at specific sites on chosen RNA transcripts (Zhang et al. 2024). We previously integrated CIP technology (Liang et al. 2011; Stanton et al. 2018; Zhao et al. 2018) with a CRISPR-based editor to achieve temporal controlled m^6^A editing (Shi et al. 2022). In this method, a chemical inducer abscisic acid (ABA) induced the heterodimerization between ABI and PYL proteins fused individually to dCas13b and a m^6^A writer or eraser, which when combined with transcript-specific guide RNAs (gRNAs), enables the inducible and reversible m^6^A editing. We further developed a CIP-mediated split dCas13b-m^6^A editing platform, which controls the assembly and disassembly of the dCas13b-editing complex to minimize the interference of the large CRISPR editing complex with the target RNA (Xu et al. 2023).

To expand the RNA modification editing toolkit for m^5^C, herein we report a cellular signal and chemical ligand controlled split dCas13b m^5^C-writing system through recruiting NSUN2 or NSUN6 to targeted RNA transcripts. In this design, half of the split dCas13b is fused to ABI and m^5^C writers, and the other half to PYL. ABA induces the heterodimerization of ABI and PYL, which leads to the reconstitution of a fully functional dCas13b-m^5^C editor at the target RNA. The process is reversible by the removal of ABA to disassemble dCas13b and release the editing complex from the transcript. Using caged ABA prodrugs, this platform can be transformed into an m^5^C editing system that is activated upon sensing specific cellular conditions. This new method expands the toolbox for programmable, temporal and cellular context-dependent manipulation of m^5^C, contributing to the study of RNA methylation biology in living cells.

## RESULTS

### Characterize the RNA substrate specificity of NSUN2 and NSUN6

NSUN2 and NSUN6 are both proven m^5^C writers but methylate distinct RNA transcripts in cells (Liu et al. 2021; Selmi et al. 2021). It has been reported that the consensus RNA sequence motif for NSUN2 is CNGGG while that of NSUN6 is CTCCA (Selmi et al. 2021). It is unclear if the distinct transcript preferences of NSUN2 and NSUN6 originate from how endogenous NSUN2 and NSUN6 are targeted in cells or from the intrinsic sequence requirement for the methyltransferase catalytic activity of NSUN2 and NSUN6. To explore the possibility of developing a universal editor that is able to use either NSUN2 or NSUN6 to install m^5^C on any chosen RNA transcripts regardless of their natural preferences, we tested whether NSUN2 and NSUN6 catalytic domains are interchangeable for m^5^C deposition on confirmed NSUN2 and NSUN6 target transcripts using dCas13b-NSUN2 or dCas13b-NSUN6 fusions (Fig. 1A). We cloned the constructs (Fig. 1B) for expressing fusion proteins linking dCas13b to the methyltransferase catalytic domains (CD) of NSUN2 (amino acid 180–430) or NSUN6 (amino acid 221–470) with an HA tag (Supplemental Table S1; Liu et al. 2017; Yang et al. 2017; Bohnsack et al. 2019). We confirmed the expression of dCas13b-NSUN2 and dCas13b-NSUN6 fusion proteins in HEK293T cells by western blotting (Supplemental Fig. S1). Previous studies have identified NSUN2-dependent m^5^C writing at the *p16* C391 site within the coding sequences (CDS) (Zhang et al. 2012; Dai et al. 2024), *p21* C2079 within its 3′ untranslated region (UTR) (Li et al. 2017), and *ERBB2* C320 in the CDS (Yuan et al. 2014; Dai et al. 2024). In contrast, the m^5^C at *NUP210* C867/869 sites in the CDS and *TIMM50* C1628 in the 3′ UTR have been reported to be installed by NSUN6 (Selmi et al. 2021). We designed and cloned sgRNAs targeting these reported m^5^C regions on *p21, p16, ERBB2, NUP210*, and *TIMM50* mRNA transcripts to recruit dCas13b-NSUN2 or dCas13b-NSUN6. We transfected sgRNAs with dCas13b-NSUN2, dCas13b-NSUN6, or dCas13b-PYL (as a negative control) into HEK293T cells for 48 h. Total RNA was extracted and m^5^C enrichment under each condition was quantified by methylated RNA immunoprecipitation (MeRIP) using anti-m^5^C antibodies followed by quantitative polymerase chain reaction (qPCR) assays. We found that NSUN2 selectively increased m^5^C levels on *p16* and *p21* but showed no activity on *NUP210* or *TIMM50* (Fig. 1C,D), whereas NSUN6 specifically edited *NUP210* and *TIMM50* without affecting *p21* or *p16* (Fig. 1F,G). Notably, both NSUN2 and NSUN6 were capable of writing m^5^C on *ERBB2* (Fig. 1E). These results suggest that NSUN2 and NSUN6 CDs are not interchangeable for m^5^C editing in most cases and an appropriate methyltransferase (NSUN2 or NSUN6) needs to be recruited based on the RNA transcript.

**FIGURE 1.**
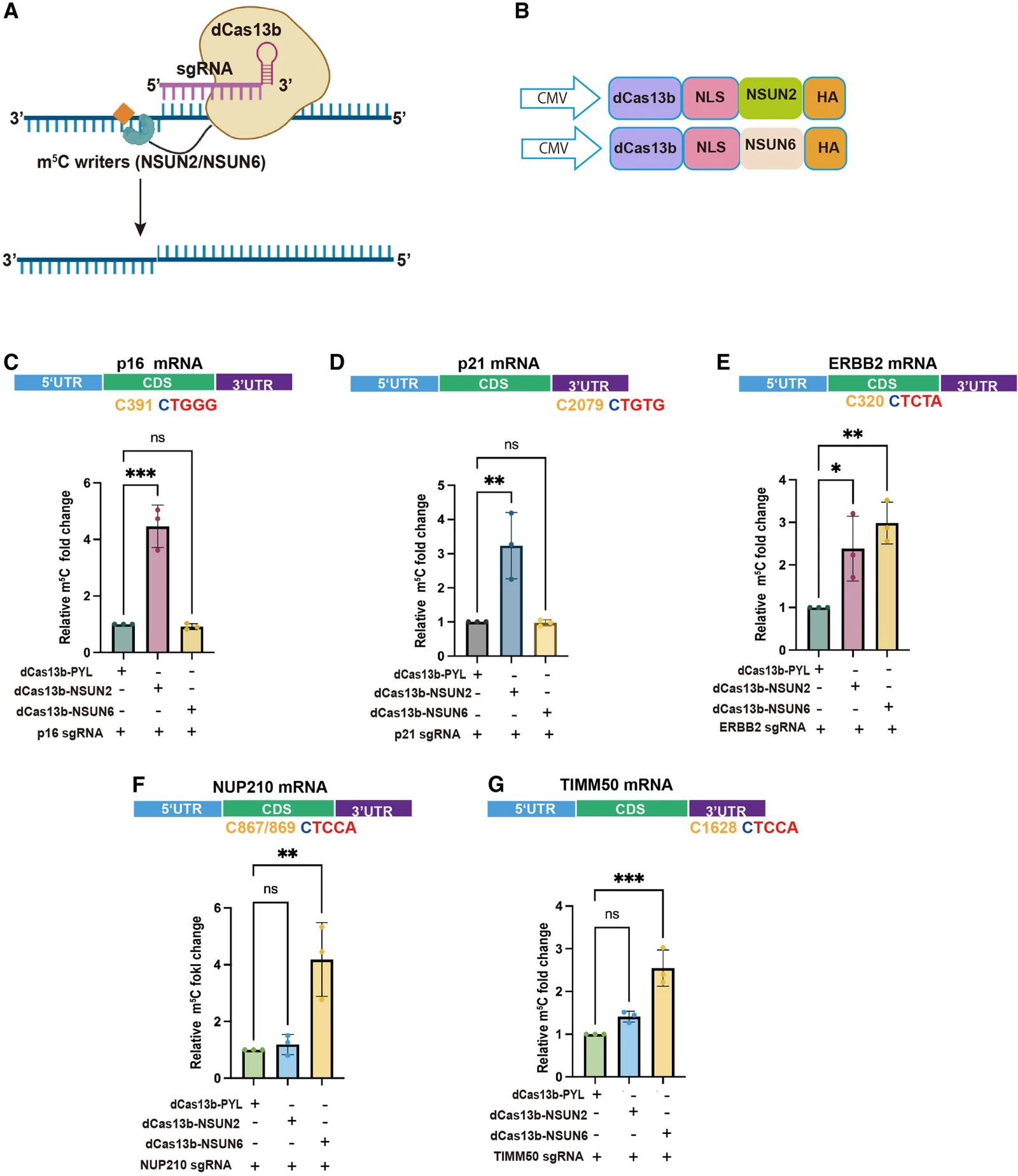
RNA substrate specificity of NSUN2 and NSUN6. (*A*) Scheme of sgRNA-guided dCas13b-NSUN2 and dCas13b-NSUN6 fusion proteins enable site-specific m^5^C writing on a target RNA transcript. (*B*) DNA constructs cloned to express dCas13b-NSUN2 and dCas13b-NSUN6. (*C*–*G*) The relative m^5^C level changes at the RNA regions of reported NSUN2 target sites *p16* C391 (*C*), *p21* C2079 (*D*), and *ERBB2* C320 (*E*), and NSUN6 target sites *NUP210* C867/869 (*F*) and *TIMM50* C1628 (*G*), after transfected with the indicated constructs in HEK293T cells for 24 h and quantified by RIP (using anti-m^5^C antibodies) and qPCR assays. Fold changes were calculated by comparing to the dCas13b-PYL condition. Error bars represent mean ± SEM from independent biological replicates (*n* = 3). Statistical significance was determined by one-way ANOVA. (ns) *P* > 0.5; (*) *P* ≤ 0.05; (**) *P* ≤ 0.01; (***) *P* ≤ 0.001.

### Engineer a split inducible m^5^C editing system

Inspired by the inducible split dCas13b-METTL3 m^6^A editing system developed in our laboratory (Xu et al. 2023), we engineered a split m^5^C writing platform with the C-terminal half of split dCas13b fused to ABI and the catalytic domain of NSUN2 or NSUN6, and the N-terminal half to PYL (Xu et al. 2023), which can be reconstituted into a functional dCas13b-NSUN2/NSUN6 editor in the presence of ABA (Fig. 2A). We cloned the plasmids expressing these fusion proteins with FLAG and HA tags included for detection purposes (Fig. 2B) and tested the expression of the newly engineered C-terminal half of split dCas13b-NSUN2/NSUN6 in HEK293T cells by western blotting (Supplemental Fig. S2). To test this m^5^C writing system, we first examined if the split dCas13b-m^5^C editing complex can be assembled at the targeted RNA sites. We transfected HEK293T cells with *p21* sgRNA plus split dCas13b-NSUN2 (C-dCas13b-NSUN2 plus N-dCas13b-PYL), *NUP210* sgRNA plus split dCas13b-NSUN6 (C-dCas13b-NSUN6 plus N-dCas13b-PYL), or *ERBB2* sgRNA plus split dCas13b-NSUN2 or NSUN6 for 24 h. dCas13b-PYL was used as a negative control, and nonsplit dCas13b-NSUN2/NSUN6 fusion as positive controls. A total of 100 μM ABA or DMSO was added to cells and incubated for another 24 h. Cells were then harvested and subjected to the crosslink immunoprecipitation (CLIP) using anti-FLAG or HA antibodies, followed by qPCR analyses, to quantify the enrichment of assembled dCas13b-m^5^C editors on the targeted RNA transcripts. m^5^C-MeRIP and qPCR assays were used to quantify m^5^C level changes on these transcripts. We observed significant increases in HA and FLAG levels on *P21* and *NUP210*, indicating the successful recruitment of both halves of the split dCas13b-NSUN2/NSUN6 editors on targeted RNA transcripts (Fig. 2C,D). We also observed that the m^5^C level on *P21, NUP210, TIMM50* and *ERBB2* transcripts increased only when ABA was added to the transfected cells with the split dCas13b-m^5^C editors and the elevated m^5^C levels were comparable to that caused by nonsplit dCas13b-NSUN2/NSUN6 fusions (Fig. 2E–H). In addition, we investigated the dosage response of ABA in inducing m^5^C writing on *ERBB2*. We observed that m^5^C level on *ERBB2* increased with ABA dosage increased. RNA and protein level levels also decreased along with ABA level increased (Supplemental Fig. S3). Taken together, these results suggested that the split-dCas13b-m^5^C writers can be reconstituted and achieve m^5^C writing on targeted RNA transcripts upon ABA addition.

**FIGURE 2.**
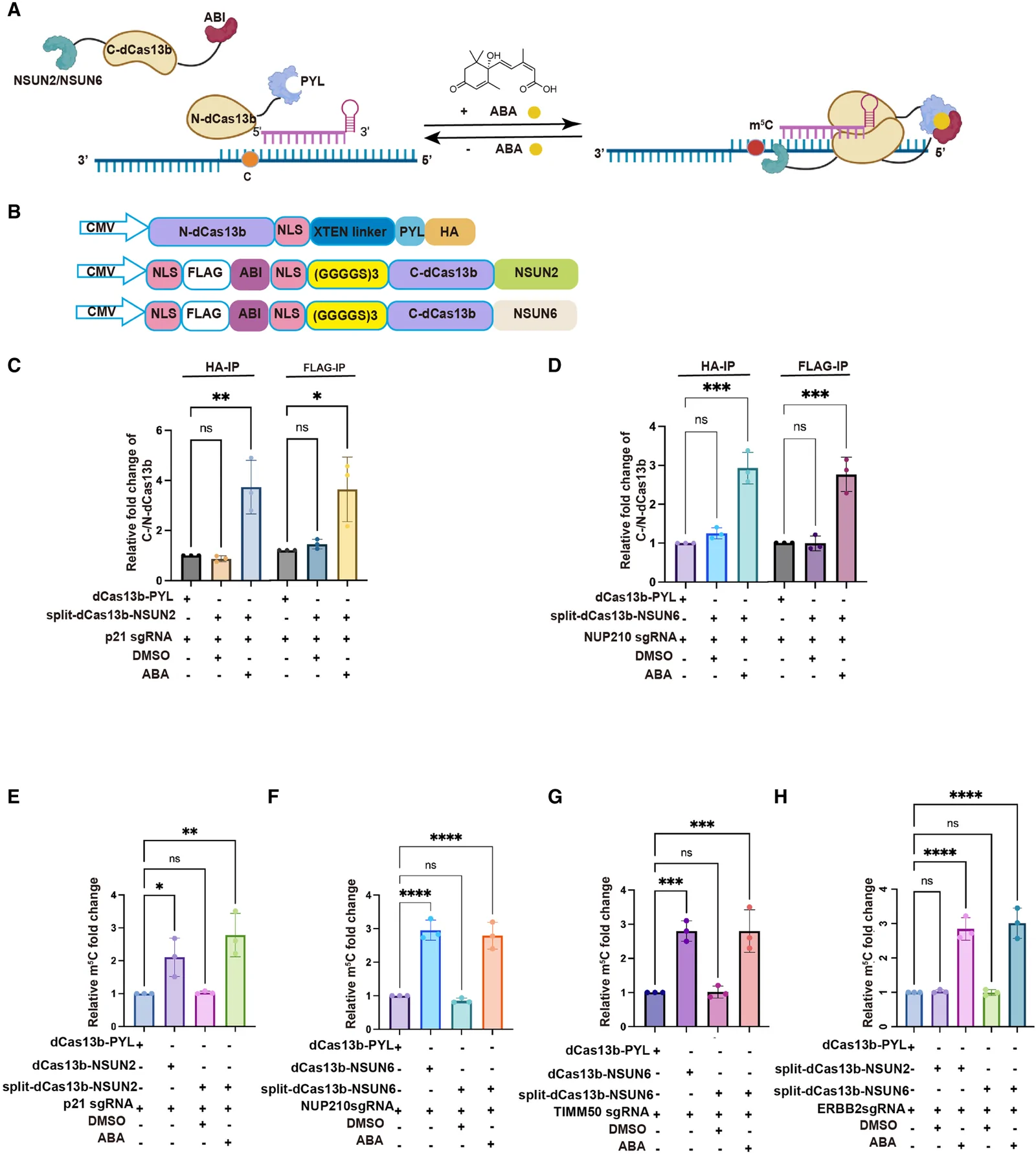
The m^5^C writing by the ABA-inducible split dCas13b-NSUN2/NSUN6 m^5^C editors. (*A*) Scheme of the inducible m^5^C editor where ABA triggers the reconstitution of a fully functional dCas13b-m^5^C editing complex from two inactive halves at the sgRNA-targeted RNA transcript. (*B*) DNA constructs cloned to express ABA-inducible split dCas13b-NSUN2 and dCas13b-NSUN6 (C-dCas13b-NSUN2/NSUN6 plus N-dCas13b-PYL). (*C*,*D*) The relative enrichment levels of the N- and C-terminal halves of the split dCas13b-NSUN2 on *p21* mRNA (*C*) or split dCas13b-NSUN6 on *NUP210* mRNA (*D*) in HEK293T cells after transfected with indicated constructs for 24 h and treated with either DMSO or ABA (100 μM) for another 24 h. Fold changes were measured by RIP (suing HA or FLAG antibodies) followed by qPCR assays and calculated by normalizing to the dCas13b-PYL condition. (*E*–*H*) The relative m^5^C level changes on indicated RNAs in the region surrounding reported m^5^C site—*p21* C2079 (*E*), *NUP210* C867/869 (*F*), *TIMM50* C1628 (*G*), and *ERBB2* C320 (*H*) in HEK293T cells after transfected with indicated constructs for 24 h and treated with either DMSO or ABA (100 μM) for another 24 h. Fold changes were measured by RIP (using anti-m^5^C antibodies) followed by qPCR assays and calculated by comparing to the dCas13b-PYL condition. Error bars represent mean *±* SEM from independent biological replicates (*n* = 3). Statistical significance was determined by one-way ANOVA. (ns) *P* > 0.5; (*) *P* ≤ 0.05; (**) *P* ≤ 0.01; (***) *P* ≤ 0.001; (****) *P* ≤ 0.0001.

### Selectivity and reversibility of the split dCas13b-m^5^C editing system

To investigate the selectivity of the split dCas13b-m^5^C editing system, we transfected HEK293T cells with split dCas13b-NSUN2 plus *p21* sgRNA, or split dCas13b-NSUN6 plus *NUP210* sgRNA, for 24 h, followed by ABA or DMSO treatment for another 24 h. dCas13b-PYL was transfected as a negative control, and nonsplit dCas13b-NSUN2/NSUN6 fusion as positive controls. Cells were harvested to quantify the m^5^C level by m^5^C-MeRIP and qPCR analyses at *p21* C2079, *NUP210* C867/869 and other reported m^5^C sites, including *p16* C391, *ERBB2* C320, and *TIMM50* C1628. We observed that only *p21* C2079 and *NUP210* C867/869 showed significant m^5^C increases when targeted by respective sgRNAs and split dCas13b-editors but not at other nontarget m^5^C sites (Fig. 3A,B), indicating that the split dCas13b-m^5^C editors were able to selectively deposited m^5^C on targeted RNA transcripts.

**FIGURE 3.**
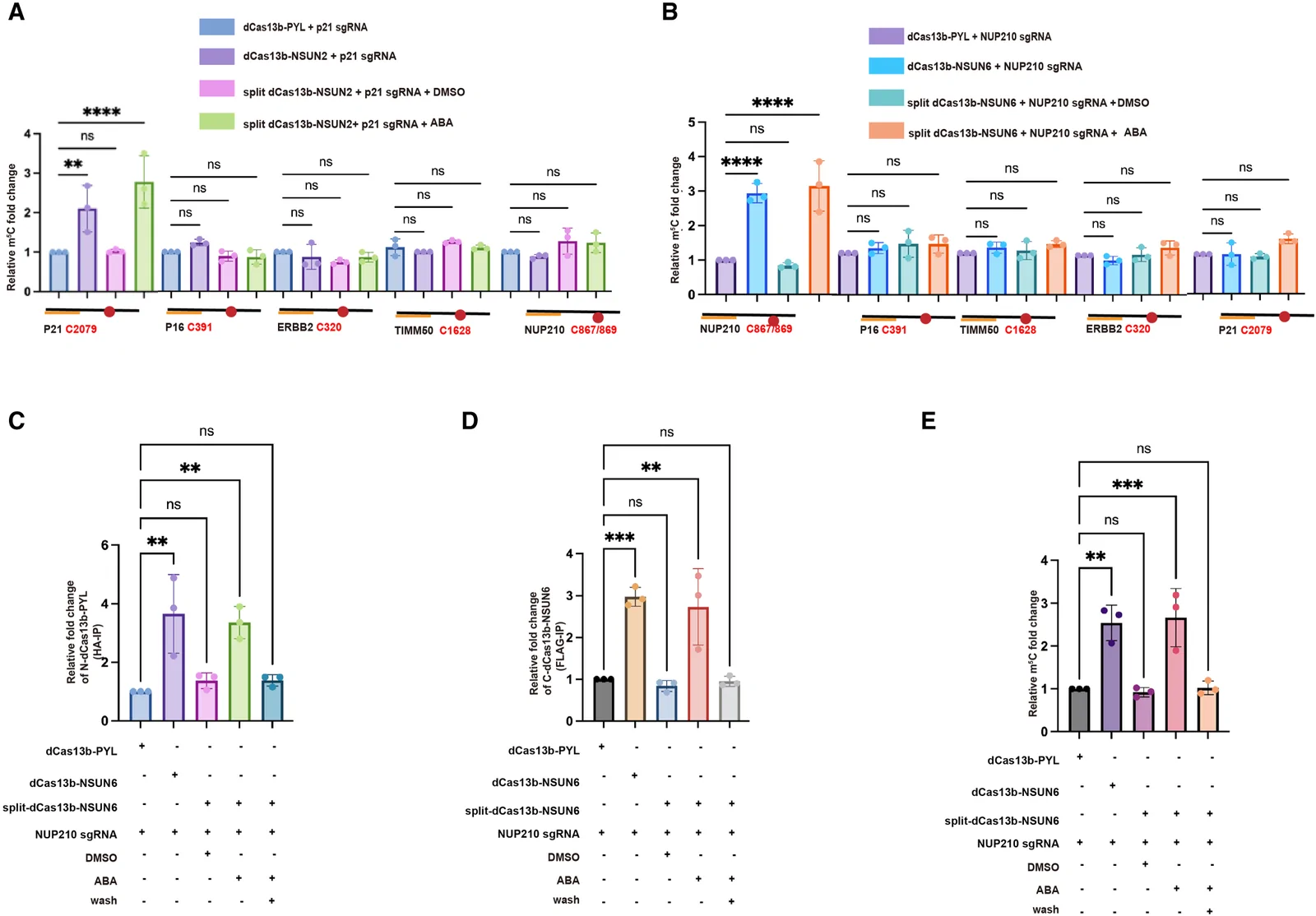
Selectivity and reversibility of the ABA-inducible split dCas13b-m^5^C writing system. (*A*) The relative m^5^C level changes on *P21* and other indicated RNAs in the regions containing the reported m^5^C site in HEK293T cells after transfected with indicated constructs and *P21*-targeting sgRNA for 24 h, and treated with either DMSO or ABA (100 μM) for another 24 h. Fold changes were measured by RIP (using anti-m^5^C antibodies) followed by qPCR assays and calculated by normalizing to the dCas13b-PYL condition. (*B*) The relative m^5^C level changes on *NUP210* and other indicated RNAs in the regions containing the reported m^5^C site in HEK293T cells after transfected with indicated constructs and *NUP210*-targeting sgRNA for 24 h, and treated with either DMSO or ABA (100 μM) for another 24 h. Fold changes were measured by RIP (using anti-m^5^C antibodies) followed by qPCR assays and calculated by normalizing to the dCas13b-PYL condition. (*C*–*E*) The relative enrichment levels of the N- and C-terminal halves of the split dCas13b-NSUN6 and m^5^C on NUP210 mRNA in HEK293T cells after transfected with indicated constructs for 24 h and treated with either DMSO or ABA (100 µM) for another 24 h. Fold changes were measured by RIP (using HA, FLAG or m^5^C antibodies) followed by qPCR assays and calculated by comparing to the dCas13b-PYL condition. Error bars represent mean *±* SEM from independent biological replicates (*n* = 3). Statistical significance was determined by one-way ANOVA. (ns) *P* > 0.5; (*) *P* ≤ 0.05; (**) *P* ≤ 0.01; (***) *P* ≤ 0.001; (****) *P* ≤ 0.0001.

To investigate the reversibility of m^5^C writing and the release of the assembled split dCas13b-m^5^C editor after ABA removal, we transfected HEK293T cells with split dCas13b-NSUN6 plus *NUP210* sgRNA for 24 h and treated cells with ABA or DMSO for another 24 h. Cells were then washed with fresh warmed media without ABA to remove ABA or not washed as a control. Cells were incubated for an additional 24 h, before cells were harvested to quantify the level changes of m^5^C and the two halves of the split dCas13b-m^5^C editor on *NUP210* mRNA using m^5^C-MeRIP/CLIP-qPCR assays. We observed that the enrichment of both N- and C-terminal halves (Fig. 3C,D), as well as the level of m^5^C on *NUP210* mRNA (Fig. 3E), decreased to the background level at 24 h after washing, confirming that the m^5^C writing is reversible and the split dCas13b-NSUN6 complex can be released from the RNA after removing ABA.

### Effects of m^5^C on edited mRNA transcripts

The effects of m^5^C on *NUP210* and *ERBB2* mRNAs as well as *TIMM50* have not been reported. Knocking down NSUN2 in HeLa cells did not influence the level of *p21* mRNA but reduced the *p21* protein level, indicating m^5^C on *p21* can enhance translation (Li et al. 2017). To examine the impact of m^5^C installed by the split dCas13b-m^5^C writers on tested mRNAs in HEK293T cells, we transfected cells with nonsplit dCas13b-NSUN2/dCas13b-NSUN6, dCas13b-PYL (as a negative control), split-dCas13b-NSUN2/NSUN6 plus sgRNA (as indicated) for 24 h. After treating cells with ABA or DMSO for another 24 h, cells were harvested and subjected to reverse transcription (RT)-qPCR assays to measure the relative mRNA abundance. We observed that the levels of both *p21* and *NUP210* mRNAs were significantly increased, while *ERBB2* and *TIMM50* mRNA levels were reduced when m^5^C was deposited on these mRNAs (Fig. 4A–D). The observed increase and decrease of the steady-state mRNA levels indicated m^5^C either reduce (stabilize) or increase (destabilize) the degradation rate of these mRNA transcripts. We also examined changes in protein levels by western blotting under these conditions. We observed increased *p21* and *NUP210* and reduced *ERBB2*, *TIMM50* protein levels when m^5^C was deposited (Fig. 4E–H), which were consistent with the changes in the mRNA level of these transcripts.

**FIGURE 4.**
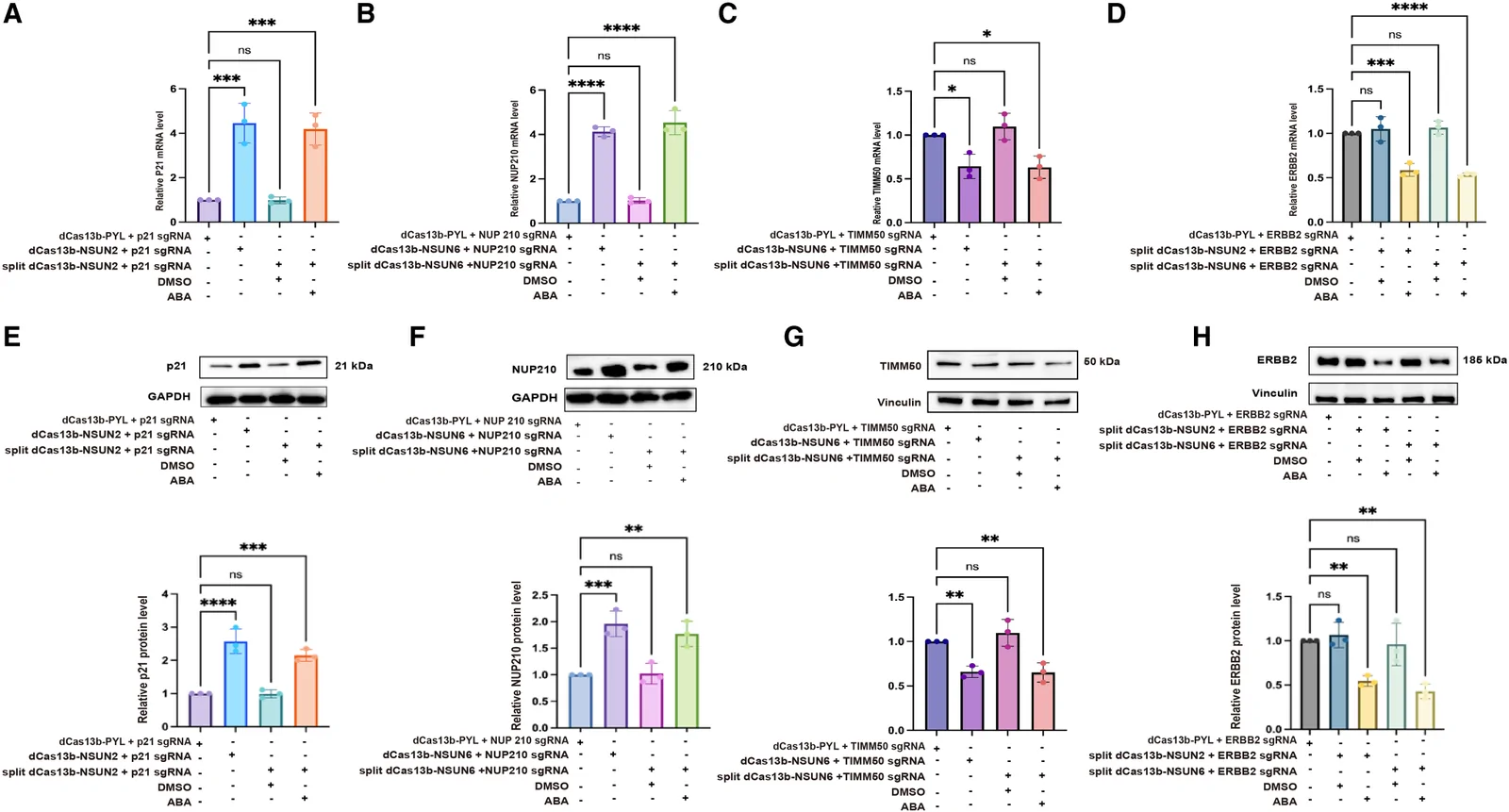
Effects of deposited m^5^C on the levels of RNA transcripts and proteins. (*A*–*D*) The relative mRNA levels of *p21* (*A*), *NUP210* (*B*), *ERBB2* (*C*), and *TIMM50* (*D*) in HEK293T cells after transfected with indicated constructs for 24 h, and treated with either DMSO or ABA (100 μM) for another 24 h. The mRNA level changes were measured by RT-qPCR assays and calculated by normalizing to the dCas13b-PYL condition. (*E*–*H*) The protein levels and changes of *p21* (*E*), *NUP210* (*F*), *ERBB2* (*G*) and *TIMM50* (*H*) in HEK293T cells after transfected with indicated constructs for 24 h, and treated with either DMSO or ABA (100 μM) for another 24 h. The protein levels were determined by western blotting. GAPDH or vinculin was used as the loading controls. (*Top* panels) The representative western blotting data. (*Bottom* panels) The quantification of the western blotting data on top. The relative protein levels shown in *A*–*H* were calculated by comparing to the condition of dCas13b-PYL. Error bars represent mean *±* SEM from independent biological replicates (*n* = 3). Statistical significance was determined by one-way ANOVA. (ns) *P* > 0.5; (*) *P* ≤ 0.05; (**) *P* ≤ 0.01; (***) *P* ≤ 0.001; (****) *P* ≤ 0.0001.

### Cellular signal-induced m^5^C writing by incorporating caged ABA prodrugs

Existing CRISPR-based transcript-specific RNA modification editing platforms are either constitutively active or under the control of a user-imposed input, such as a chemical ligand or light (Shi et al. 2022; Xu et al. 2023; Xie et al. 2024). It will be invaluable to establish editing tools that can sense local cellular or disease environments and trigger the editing of specific RNA transcripts correspondingly in a preprogrammed manner for desired biological or potential therapeutic effects. To test this possibility, we investigated the conditional m^5^C writing induced by a hypoxia- or senescence-associated signal, using the split dCas13b-m^5^C writers combined with caged ABA prodrugs.

Aberrant m^5^C methylation has been recognized as a critical epitranscriptomic hallmark of tumorigenesis (Nombela et al. 2021; Zhang et al. 2021; Song et al. 2022; Gu et al. 2023). Dysregulation of m^5^C methyltransferases leads to a global reduction of m^5^C, which disrupts posttranscriptional gene control and facilitates oncogenic transformation (Yang et al. 2021; Hu et al. 2023) Targeting m^5^C modification mechanisms in tumor cells has emerged as a new strategy for cancer therapies (Chen et al. 2025; Yuan et al. 2025). Hypoxia is a hallmark of tumor microenvironments (TME) in many solid tumors (Harris 2002; Liao et al. 2023), and a hypoxia-triggered m^5^C writing platform may mitigate the m^5^C reduction on critical tumor-associated genes in cancer cells. We have previously reported several caged ABA molecules that remain inactive for triggering CIP events until the presence of specific cellular or disease signals that catalyze the uncaging reaction and regenerate active ABA (Wright et al. 2015; Zeng et al. 2015, 2017; Chen et al. 2023; Nguyen et al. 2024, 2025). For example, an ABA prodrug 1, has been developed to respond to the nitroreductase (NTR) overexpressed under the hypoxic condition in TME and has been used to conditionally control the chimeric antigen receptor (CAR)-T cell activity in TME (Nguyen et al. 2025).

To achieve hypoxia-induced m^5^C writing, we incorporated ABA prodrug 1 into our split dCas13b-m^5^C writing system (Fig. 5A). In the presence of NTR, we expect that ABA prodrug will undergo a nitro reductase-mediated reduction on the caging group, which can subsequently release the active ABA inducer to assemble the dCas13b-m^5^C editor and achieve m^5^C writing. *TIMM50* mRNA was selected as the target transcript to test NTR-triggered m^5^C editing. We transfected HEK293T cells with split dCas13b-NSUN6 plus *TIMM50* sgRNA for 24 h. Cells were then treated with DMSO, ABA, precleaved ABA prodrug 1 (1 pretreated with NTR before adding to cells) (Nguyen et al. 2025), or ABA prodrug 1 with or without NTR added to cells, for another 24 h. Cells were then harvested to quantify the m^5^C level on *TIMM50* by m^5^C-RIP and qPCR analyses. We observed that m^5^C increased only when cells were treated with ABA, or ABA prodrug 1 incubated with NTR before or during cell culture (Fig. 5B), supporting that a signal-triggered m^5^C editing system can be created by incorporating specific ABA prodrugs.

**FIGURE 5.**
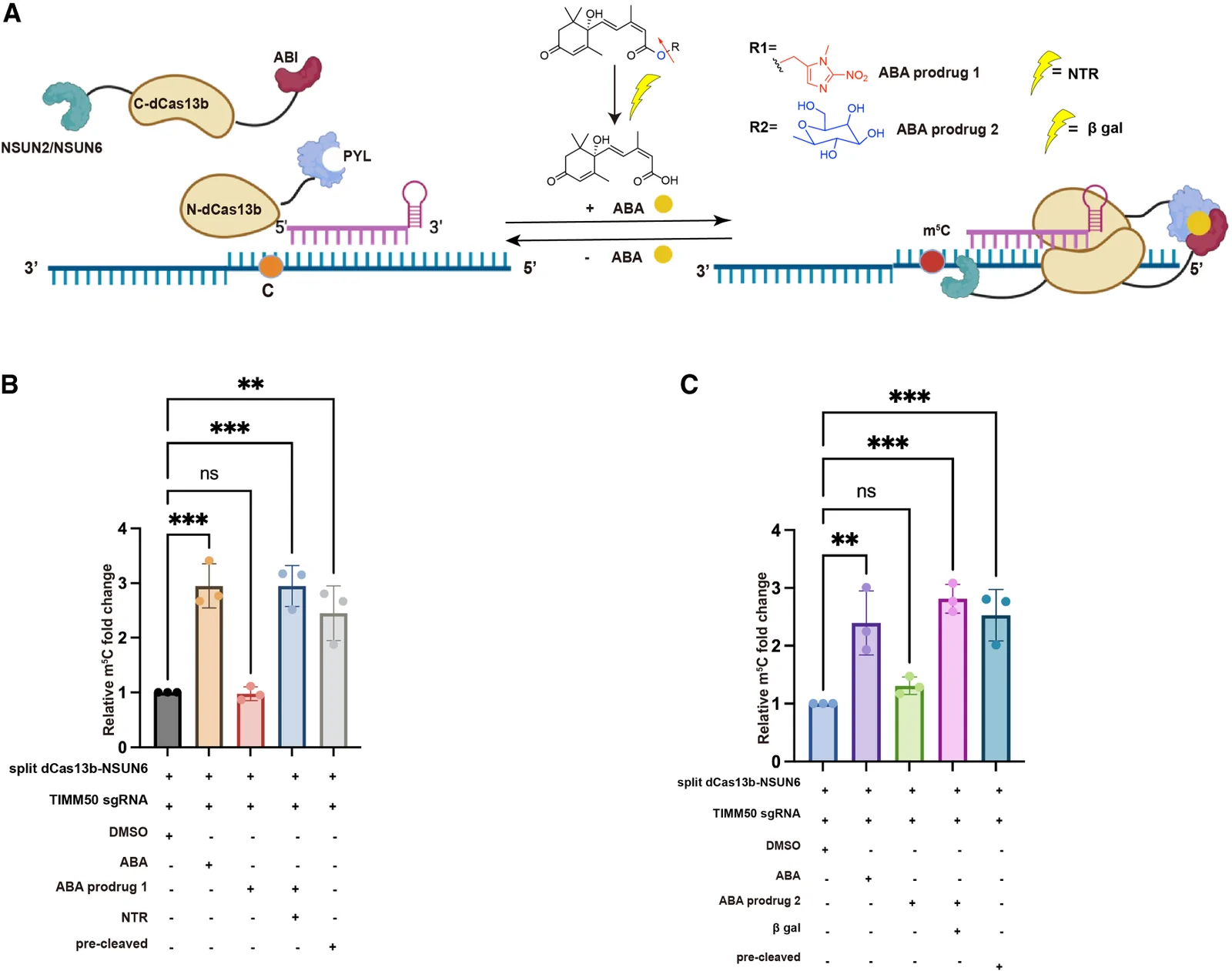
Signal-induced m^5^C editing by incorporating ABA prodrugs. (*A*) Scheme of the signal-induced m^5^C editing system using ABA prodrugs and the split dCas13b-m^5^C editors. ABA prodrugs are designed to be uncaged in response to hypoxia or senescence-associated signals, releasing active ABA to dimerize the split dCas13b-NSUN2/NSUN6 halves and achieve m^5^C writing at target mRNAs. (*B*,*C*) The relative m^5^C fold changes on *TIMM50* mRNA in HEK293T cells under the indicated treatment conditions using hypoxia/NTR-responsive ABA prodrug 1 (*B*) or senescence/β-gal-responsive ABA prodrug 2 (*C*). Cells were first transfected with constructs expressing the split dCas13b-NSUN6 and *TIMM50* sgRNAs for 24 h, followed by treating with DMSO, ABA (100 μM), ABA prodrug 1 or 2 (100 μM), or precleaved ABA prodrug 1 or 2 (100 μM) for another 24 h. ABA prodrug 1 was precleaved by NTR. ABA prodrug 2 was precleaved by β-gal. m^5^C fold changes were measured by RIP (using m^5^C antibodies) followed by qPCR assays and calculated by comparing to the DMSO-treated samples. Error bars represent mean *±* SEM from independent biological replicates (*n* = 3). Statistical significance was determined by one-way ANOVA. (ns) *P* > 0.5; (*) *P* ≤ 0.05; (**) *P* ≤ 0.01; (***) *P* ≤ 0.001; (****) *P* ≤ 0.0001.

To further explore the general applicability of this strategy, we tested the induced m^5^C writing triggered by the cellular senescence condition. Downregulation of NSUN2 and NSUN6 has been shown to reduce m^5^C across transcriptome and result in senescence-associated phenotypes (Schosserer et al. 2015; Zhou et al. 2025) β-galactosidase (β-gal), a lysosomal glucosidase, is widely recognized as a hallmark biomarker overexpressed in senescent cells (Lee et al. 2014; Li et al. 2020b). Incorporating another ABA prodrug 2, which was previously developed to be activated by β-gal (Nguyen et al. 2024), we tested a β-gal inducible m^5^C writing system using *TIMM50* as the target transcript. HEK293T cells were transfected with split dCas13b-NSUN6 plus *TIMM50* sgRNA for 24 h, followed by the treatment with DMSO, ABA, precleaved ABA prodrug 2 (2 pretreated with β-gal before adding to cells) (Nguyen et al. 2024) or ABA prodrug 2 in the presence or absence of β-gal in cell culture, for another 24 h. Cells were then harvested and the m^5^C levels on *TIMM50* under each condition was quantified by m^5^C-RIP and qPCR assays. We observed increased m^5^C levels on *TIMM50* when cells were treated with ABA or ABA prodrug 2 treated with β-gal before or during cell culture (Fig. 5C). These results demonstrate that m^5^C editing can be triggered by different cellular or disease-associated signals by integrating tailored ABA prodrugs into the split dCas13b-m^5^C writing system.

## DISCUSSION

Growing interest in the RNA epigenetics field has motivated the development of CRISPR/dCas-based RNA modification editing technologies for the precise, transcript-specific manipulation of chosen RNA modifications. Although several methods to achieve the writing and erasing of m^6^A have been reported, tools enabling programmable and temporally controlled m^5^C editing are still lacking. In this study, we developed a conditional m^5^C-writing technology, inducible by chemical inducer or cellular signals. Reported m^5^C writers NSUN2 and NSUN6 show distinct sequence motif preferences in cells. We questioned whether the artificially targeting of methyltransferase catalytic domain of either writer to chosen RNA transcripts by the CRISPR method could be sufficient to deposit m^5^C by either writer domain. To answer this, we used dCas13b-NSUN2 and dCas13b-NSUN6 and sgRNAs to individually target NSUN2 and NSUN6 methyltransferase domain to five endogenous mRNA transcripts known to either be the substrate of NSUN2 (*p16*, *p21*, and *ERBB2*) or NSUN6 (*NUP210* and *TIMM50*) in cells. We found that, among the tested transcripts, most transcripts can indeed only be edited by either NSUN2 or NSUN6 (Fig. 1C,D,F,G). Interestingly, we found that *ERBB2*, previously reported to be edited by NSUN2, can also be edited by NSUN6 (Fig. 1E). Sequence analysis revealed that the local context of the *ERBB2* m^5^C site (*C*TCTA) closely resembles the NSUN6 canonical recognition motif (*C*TCCA), differing only at position +3 (C→T)—a substitution previously shown to significantly impair endogenous NSUN6 activity (Yang et al. 2021). Despite this suboptimal sequence context, dCas13-NSUN6 retained the ability to install m^5^C at this locus, suggesting that sgRNA-guided proximity recruitment may compensate for reduced intrinsic substrate affinity. In contrast, NSUN2 exhibits a structural preference for hairpin-proximal substrates (Selmi et al. 2021), consistent with the presence of a hairpin structure near the *ERBB2* m^5^C site. This suggests that dCas13-NSUN2 editing at this locus may benefit from both proximity-driven recruitment and structure-assisted recognition. These results indicate that NSUN2 and NSUN6 have their own sequence motif requirement for most targets and forced recruitment of these enzyme domains to sites without suitable sequence matching will not lead to m^5^C deposition. As a result, NSUN2 and NSUN6 compatibility with individual RNA substrates should be considered when extending this approach to edit other RNA transcripts. This observation also suggests that any off-site targeting of NSUN2 and NSUN6 by CRISPR methods will not necessarily result in m^5^C editing if no matching cytosine-containing sequence motifs of NSUN2/NSUN6 are in range. One point worth noting is that only a limited number of RNA transcripts were tested in this study and whether the observation can be extended and applied across the transcriptome remains to be determined.

Informed by the results, we engineered both NSUN2 and NSUN6 versions of inducible m^5^C editing platforms based on a split dCas13b system containing two inactive halves that can be assembled into a functional dCas13b complex in the presence of the inducer ABA. We showed that the ABA-inducible m^5^C writing using this split dCas13b system can be achieved in a similar efficiency as the nonsplit dCas13b-NSUN2/NSUN6 fusions (Fig. 2) and confirmed that the assembly of the split dCas13b complex and m^5^C writing are reversible and selective among tested RNA transcripts (Fig. 3). The transcriptome-wide editing specificity based on dCas13-guide m^6^A writer has been reported showing the off-site editing on ∼3.5% transcripts in the whole transcriptome (Wilson et al. 2020). With the stringent sequence requirement of NSUN2 and NSUN6, we expect that the transcriptome-wide editing specificity of our m^5^C editing system may be comparable or lower than the previous report. In addition, the implementation of inducer-controlled conditional editing mechanism will enhance the temporal specificity that can limit undesired effects from off-site targeting, compared to the constitutively active editing systems. However, transcriptome-wide analyses using next generation sequencing or other methods will need to be performed in the future to rigorously evaluate the off-target editing of this inducible m^5^C editing system. In addition, while our targeted qPCR-based analyses indicate that m^5^C editing is selective on the tested transcripts, we have not performed single-base resolution sequencing analyses to confirm the precise m^5^C editing site on each transcript. However, the m^5^C sites on RNA transcripts chosen for editing in this study have been confirmed from previous studies, which also revealed only one m^5^C site on *P16*, *P21*, *ERBB2*, and *TIMM50*, and two adjacent sites on *NUP210* (as shown in Fig. 1C) (Zhang et al. 2012; Li et al. 2017; Selmi et al. 2021; Dai et al. 2024). As a result, we reason that any m^5^C change within the probed region on each tested RNA transcript should be expected to occur on the reported methylation site.

We investigated the functional consequences of m^5^C deposition by the split dCas13b-m^5^C writers on *p21* (within 3′UTR), *NUP210* (within CDS) and *ERBB2* (within CDS) in HEK293T cells. We observed that m^5^C increased the steady state mRNA levels of *p21* and *NUP210*, while reduced the mRNA level of *ERBB2* (Fig. 4), which suggested that m^5^C influenced the stability of these mRNA transcripts. These results also demonstrated the context-dependent effects of m^5^C, which varied depending on the sites and the RNA transcripts. Although the functions of m^5^C on *NUP210* and *ERBB2* have not been reported, it has been shown that, in HeLa cells, an increased m^5^C at 3′ UTR of *p21* did not change its mRNA level but instead increasing *p21* protein expression (Li et al. 2017), suggesting that m^5^C influence *p21* translation instead of mRNA stability. The observed differences in biological effects of m^5^C at *p21* 3′ UTR in different cell types further illustrates the cellular context-dependency of m^5^C's functions. Alternatively, the differences may also arise from the different methods used to manipulate the m^5^C levels in these studies (i.e., globally knocking down NSUN2 vs. transcript-specific editing).

To explore the feasibility of a new strategy that enables the cellular context-dependent m^5^C editing, we incorporated caged ABA prodrugs into the split dCas13b-m^5^C editing system. Several ABA prodrugs have been developed to respond to diverse cellular and disease-associated signals and mediated various downstream effects (Wright et al. 2015; Zeng et al. 2015, 2017; Chen et al. 2023; Nguyen et al. 2024, 2025). Using ABA prodrugs responsive to hypoxia and senescence-associated signals, respectively, we demonstrated that conditional m^5^C deposition can be triggered under these distinct cellular conditions. We expect diverse ABA prodrugs responding to different physiological conditionals can be developed and incorporated into this inducible RNA modification editing strategy to regulate RNA transcripts in biological context-dependent manners. Collectively, this new inducible m^5^C editing strategy demonstrated in this study expands the current CRISPR-based RNA modification editing toolbox and provides a versatile platform for m^5^C studies. We envision that by expanding the effector domain repertoire and inducer chemistry, new programmable epitranscriptome editing tools to edit diverse RNA modifications at chosen RNA transcripts under specific cellular environment can be established to facilitate RNA epigenetic research. Given the reported connections of m^5^C to multiple human diseases including cancers, neurological and developmental disorders, and cardiovascular and metabolic diseases (Martinez et al. 2012; Lu et al. 2024; Guan et al. 2025; Zheng et al. 2026), this new m^5^C editing platform can contribute to the understanding of m^5^C's in these diseases and potentially lead to the development of new therapeutic strategies.

## MATERIALS AND METHODS

### Plasmid construction

Direct fusion m^5^C plasmids (dCas13b-NSUN2, dCas13b-NSUN6) were constructed by replacing METTL3 (M3) from pCMV-dCas13b-M3nls (Addgene 155366) with catalytical domain of NSUN2/NSUN6. NSUN2 and NSUN6 fragments were amplified from NSUN2 (Addgene 155793) and NSUN6 (Addgene 188060) with PCR primes that were ordered from Thermo Fisher Scientific. The CD sequence of NSUN2 and NSUN6 are listed in Supplemental Table S1, and all the PCR primers sequence are listed in Supplemental Table S2. The PCR fragments were then inserted into linearized pCMV-dCas13b-M3nls through infusion reaction (TAKARA 638952). The guided RNAs were designed through the website: https://cas13design.nygenome.org and cloned via the golden-gate cloning method by inserting the target sequence into the guide RNA backbone, pC0043-PspCas13b-crRNA (Addgene 103854). All the guide RNA spacer sequences are listed in Supplemental Table S1. All the plasmids were verified with the Sanger sequence (GENEWIZ Inc.).

### Cell culture and transfection

HEK293T cells were gifts from Dr. Gerald Crabtree at Stanford University Medical School and were cultured in Dulbecco's Modified Eagle Medium (Thermo Fisher Scientific 11965118), with the addition of 10% fetal bovine serum (Thermo Fisher Scientific A4766801) and 1% penicillin/streptomycin (Sigma-Aldrich P4333). HEK293T cells were incubated at 37°C with 5% CO_2_. Different cell passages were used for each biological replicate.

### Western blotting

HEK293T cells were seeded in a 6 well plate and transfected with 1.5 µg of each plasmid at 75% confluency. After 24 h, cells were washed with ice-cold phosphate-buffered saline (PBS) twice and lysed with 200 μL of ice-cold RIPA buffer (Thermo Fisher Scientific 89901) supplemented with 2 μL protease inhibitor cocktail (Thermo Fisher Scientific 78430). Twenty micrograms of lysate from each sample was then denatured at 95°C for 5 min and loaded into each well of SDS-PAGE gel. After separating proteins by running gel at a constant current of 100 V for 100 min, proteins were transferred from gel to the membrane at a constant current of 200 mA for 2 h at 4°C. The membrane was blocked with 5% milk in TBST (TBS + 0.5% Tween-20) at room temperature for 1 h and incubated with HA Tag Monoclonal Antibody (2-2.2.14) (Invitrogen 26183, 1:2000 dilution), FLAG Tag Monoclonal Antibody (M2) (Sigma F1804, 1:2000 dilution), or anti-GAPDH antibody (D16H11) XP Rabbit mAb (Cell Signaling Technology 5174, 1:2000 dilution) at 4°C overnight. After washing with TBST three times, the membrane was incubated with anti-rabbit IgG HRP-linked antibody (Cell Signaling Technology 7074, 1:2500 dilution) or anti-mouse IgG HRP-linked antibody (Cell Signaling Technology 7076, 1:2500 dilution) at room temperature for 1 h. Finally, the membrane was washed with TBST three times and then imaged on the ChemiDoc MP Image System (Bio-Rad Laboratories). *GAPDH* was used as the reference control.

### RNA immunoprecipitation (RIP) for m^5^C enrichment

HEK293T cells were seeded in a 10 cm dish and incubated for 16–20 h to reach ∼80% confluency. After this, cells were transfected with 6 μg of N-dCas13b-PYL plasmid and 6 μg of ABI-C-dCas13b-NSUN2 or ABI-C-dCas13b-NSUN6 plasmid and 4 μg of PspCas13b guide RNA using Lipofectamine 2000 (Thermo Fisher Scientific 11668019) according to the manufacturer's protocol. Spacer sequences of PspCas13b guide RNA are listed in Supplemental Table S1. For control groups, only 6 μg of dCas13b-PYL, dCas13b-NSUN2, or dCas13b-NSUN6 plasmid were transfected with 4 μg of the related PspCas13b guide RNAs. Unless otherwise noted, after 24 h posttransfection, cells were treated with 100 μM of ABA or DMSO in each inducible system group and incubated for another 24 h before harvest.

After 48 h posttransfection, cells were washed with PBS twice. Total RNA was isolated with TRIzol reagent (Thermo Fisher Scientific 15596026) and purified according to the manufacturer's protocol. Subsequently, the total RNA was fragmented in freshly prepared RNA fragmentation buffer (Bio-Rad E6150S) at 94°C for 1 min. The fragmented RNAs were collected and purified using RNA Clean & Concentrator kits (Zymo Research) according to the manufacturer's protocol. A portion of fragmentation RNA was saved as input samples for RT-qPCR.

For each reaction, 50 µg of total RNA was incubated with 5 µL of 5-methylcytosine (m^5^C) antibody (Active Motif MSPP-396492) in 250 µL of reaction buffer (150 mM NaCl, 10 mM Tris-HCl, pH 7.5, 0.1% NP-40 in nuclease-free H_2_O), supplemented with 5 µL of RNasin Plus inhibitor (Promega N2611) for 1 h at 4°C with orbital rotation. Protein G magnetic beads (25 µL per reaction; NEB S1430) were prewashed twice with 200 µL of reaction buffer and subsequently added to the RNA–antibody mixture, followed by incubation for 2 h at 4°C with rotation. The beads were sequentially washed twice with 200 µL of reaction buffer, twice with 200 µL of low-salt buffer (50 mM NaCl, 10 mM Tris-HCl, pH 7.5, 0.1% NP-40), and twice with 200 µL of high-salt buffer (500 mM NaCl, 10 mM Tris-HCl, pH 7.5, 0.1% NP-40). Bound RNA was eluted with 30 µL of buffer RLT (Qiagen 79216) for 1 min at room temperature.

To purify the RNA, 20 µL of Dynabeads MyOne Silane (Life Technologies 37002D) prewashed with 100 µL of buffer RLT and resuspended in 30 µL of buffer RLT were added to the eluate. Subsequently, 60 µL of absolute ethanol was mixed with the RNA–Dynabeads suspension and incubated for 1 min at room temperature. The beads were washed twice with 200 µL of 70% ethanol and air-dried for 5 min at room temperature. Purified RNA was eluted with 20 µL of RNase-free water (Gibco) by incubation for 1 min at room temperature. The recovered RNA, together with 1 µg of input total RNA, was used for cDNA synthesis and RT-qPCR analysis. The m^5^C level at target site was also detected using the corresponding qPCR primers. The primers are listed in Supplemental Table S2.

### CLIP for N-dCas13b-PYL, ABI-C-dCas13b-NSUN2 or ABI-C-dCas13b-NSUN6 enrichment

HEK293T cells were seeded into a 6 well plate and incubated for ∼18–20 h to reach 80% confluency. The cells were transfected with 1.2 μg of N-dCas13b-PYL plasmid and 1.2 μg of ABI-C-dCas13b-NSUN2 or ABI-C-dCas13b-NSUN6 plasmid and 0.8 μg of guide RNA. After 24 h posttransfection, 100 μM ABA or DMSO was added and cells were incubated for another 24 h. For ABA washout experiments, cells were washed with PBS three times and incubated with fresh DMEM (10% FBS) for another 24 h. Cells were then digested and resuspended in 1 mL DMEM and fixed with 1% formaldehyde of 27 μL (Stock 37%, Sigma F8775) for 10 min at room temperature. The cross-linking reaction was quenched by adding 50 μL of 2.5 M glycine (Fisher Bioreagents BP381-1). The cells were then centrifuged at 2500 rpm for 5 min, followed by washing with 1 mL cold PBS for three times. The supernatant was discarded, and the cell pellet was resuspended in 200 μL lysis buffer (50 mM Tris-HCl pH 7.4 150 mM NaCl, 1 mM EDTA, 0.5% NP-40, Protease inhibitor, RNase inhibitor) and lysed on ice for 30 min. The cell lysates were sonicated for 2 min (30 sec/on and 30 sec/off cycles) and centrifuged at 12,500 rpm for 10 min at 4°C. A 50 μL aliquot of supernatant was reserved as input, stored at −80°C, while 150 μL was used for immunoprecipitation with the anti-HA or anti-FLAG antibody.

For the immunoprecipitation, firstly, 2 μg anti-HA antibody (Invitrogen 26183) or anti-FLAG antibody (Sigma F1804) was added to the supernatant, then rotated at 4°C for 2 h. Then, 20 μL prewashed protein G magnetic beads (Thermo Fisher Scientific 10004D) were added to the bound mixture and incubated on the rotator at 4°C for 1 h. The bound fractions were then washed three times with 1 mL precold wash buffer (50 mM Tris-HCl pH 7.4, 150 mM NaCl, 1 mM EDTA, 0.1% TritonX-100, Protease inhibitor, and RNase inhibitor), and proteins were digested by 3 µL Proteinase K (New England Biolabs) supplemented with 50 μL reverse cross-link buffer (10 mM Tris-HCl pH 8.0, 100 mM NaCl, 1 mM EDTA, 0.5% SDS, and RNase inhibitor), then incubated at 65°C for 2 h. RNA samples were purified using RNA Clean & Concentrator-5 kits (Zymo Research) and transcribed into cDNA for qPCR analysis to determine the HA and FLAG enrichment. The primers are listed in Supplemental Table S2.

### m^5^C erasing on target gene and other nontarget RNAs

HEK293T cells were seeded in a 10 cm dish and incubated for 16–20 h to reach ∼80% confluency. After this, cells were transfected with 6 μg of N-dCas13b-PYL plasmid and 6 μg of ABI-C-dCas13b-NSUN2 or ABI-C-dCas13b-NSUN6 plasmid and 4 μg of PspCas13b guide RNA (p21 sgRNA or NUP210 sgRNA) using Lipofectamine 2000 (Thermo Fisher Scientific 11668019), according to the manufacturer's protocol. The cells were harvested for RNA extraction after treatment of 100 μM ABA or DMSO for 24 h, and the MeRIP-qPCR assay will be used to quantify the relative m^5^C level on p21 or NUP210 mRNA of target sites or nontargeted RNA loci. The primers are listed in Supplemental Table S2.

### m^5^C impact on protein levels of target genes

HEK293T cells seeded in a 12 well plate were transfected with different components of the split m^5^C editing system. After 24 h incubation, 100 μM ABA or DMSO was added to the cell and incubated for another 24 h before harvest. Proteins were extracted as mentioned above and 20 μg of whole protein were loaded for protein gel electrophoresis. p21, NUP210, ERBB2 and internal control proteins, including GAPDH and Vinculin, were probed with the corresponding anti-p21 antibody (12D1) (Cell Signaling Technology 2947, 1:1000 dilution), anti-NUP210 (E9A5G) (Cell Signaling Technology 50047, 1:1000 dilution) anti-ERBB2 (Cell Signaling Technology 2242, 1:1000 dilution), ant-GAPDH antibody (D16H11) (Cell Signaling Technology 5174, 1:2000 dilution), anti-Vinculin (E1E9V, 1:1000) (Cell Signaling Technology 13901, 1:1000 dilution) and corresponding secondary antibodies as described above.

### m^5^C impact on mRNA stability of target genes

HEK293T cells were transfected with different components of the split-m^5^C editing system, followed by adding ABA for 24 h before cells were harvested. After washing cells with PBS twice, total RNA was isolated by TRIzol reagent (Thermo Fisher Scientific 15596026) and purified according to the manufacturer's protocol. After reverse transcription, mRNA levels of target transcripts were analyzed by qPCR. GAPDH was used as the internal control for target mRNA analysis. All the primers for mRNA level qPCR analysis are listed in Supplemental Table S2.

### Hypoxia-induced m^5^C editing

One hundred micromolars of ABA-prodrug 1 were preincubated with 50 μg/mL NTR (Sigma-Aldrich) and 1 mM NADPH (Roche) at 37°C for 1 h and referred to as precleaved ABA prodrug 1. HEK293T cells were seeded in a 10 cm dish and incubated to reach ∼80% confluency. After this, cells were transfected with 6 μg of N-dCas13b-PYL-HA plasmid, 6 μg of ABI-C-dCas13b-NSUN6 plasmid, and 4 μg of TIMM50 guide RNA using Lipofectamine 2000 (Thermo Fisher Scientific 11668019), according to the manufacturer's protocol. At 24 h after plating, cells were treated with different ABA conditions, including 100 μM ABA, 100 μM ABA-prodrug 1 with or without 50 μg/mL NTR and 1 mM NADPH or precleaved ABA for 24 h before cells were harvested. After washing the cells with PBS twice, the total RNA was extracted with TRIzol reagent (Thermo Fisher Scientific 15596026) and m^5^C-IP qPCR analysis was performed.

### Senescence-induced m^5^C editing

One hundred micromolars of ABA-prodrug 2 was preincubated with 75 μM gal (Sigma-Aldrich) at 37°C for 1 h and referred to as precleaved ABA prodrug 2. HEK293T cells were seeded in a 10 cm dish and incubated to reach ∼80% confluency. After this, cells were transfected with 6 μg of N-dCas13b-PYL-HA plasmid, 6 μg of ABI-C-dCas13b-NSUN6 plasmid, and 4 μg of TIMM50 guide RNA using Lipofectamine 2000 (Thermo Fisher Scientific 11668019), according to the manufacturer's protocol. At 24 h after plating, cells were treated with different ABA conditions including 100 μM ABA, 100 μM ABA-prodrug 2 with or without 75 μM gal, or precleaved ABA for 24 h before cells were harvested. After washing the cells with PBS twice, the total RNA was extracted with TRIzol reagent (Thermo Fisher Scientific 15596026) and m^5^C-IP qPCR analysis was performed.

### RT-qPCR

The input RNA samples saved above and RNA after IP samples were used as templates for reverse transcription to synthesize cDNA according to the manufacturer's protocol (iScript cDNA Synthesis kit, Bio-Rad). The synthesized cDNAs from input and IP samples were mixed with Power SYBR Green PCR Master Mix (Applied Biosystems) for qPCR in a 384 well plate in a 7900HT Fast Real-Time PCR System (Applied Biosystems). Primers for qPCR are listed in Supplemental Table S2. All measurements were replicated in a triplet. *Ct* values were obtained from the SDS software (Version 2.4, Applied Biosystems). Folds of m^5^C level or split versions were first normalized to input and then to experimental controls and calculated according to the 2^−ΔΔ*Ct*^ method (Livak and Schmittgen 2001).

### Statistics and reproducibility

Information regarding error bars, numbers of replicates or samples, and statistical analyses is included in the corresponding figure legends. Representative results of at least three independent experiments are shown, unless otherwise indicated.

## DATA DEPOSITION

The authors declare that all data supporting the findings of this study are available within the paper and its Supplemental Material.

## SUPPLEMENTAL MATERIAL

Supplemental Material is available for this article.
